# Validation data for the quantification of the Annonaceous acetogenin annonacin in Rat brain by UPLC-MS/MS

**DOI:** 10.1016/j.dib.2016.04.067

**Published:** 2016-05-04

**Authors:** Natacha Bonneau, Isabelle Schmitz-Afonso, Alain Brunelle, David Touboul, Pierre Champy

**Affiliations:** a Laboratoire de Pharmacognosie, BioCIS, Univ. Paris-Sud, CNRS, Université Paris-Saclay, UFR Pharmacie, 5 rue J.-B. Clément, 92290 Châtenay-Malabry, France; b Institut de Chimie des Substances Naturelles, CNRS UPR2301, Université Paris-Saclay, Avenue de la Terrasse, 91198 Gif-sur-Yvette Cedex, France

**Keywords:** AAG, Annonaceous acetogenin, amu, atomic mass unit, EMA, European Medicines Agency, IS, internal standard, LLOQ, lower limit of quantification, MF, matrix factor, MQC, medium concentration quality control, QC, quality control, RSD, relative standard deviation, SRM, selected reaction monitoring, ULOQ, upper limit of quantification, UPLC-MS/MS, ultra-performance liquid chromatography tandem mass spectrometry

## Abstract

Annonaceous acetogenins (AAGs) are environmental neurotoxins from the fruit pulp of several Annonaceae species, whose consumption was linked to the occurrence of sporadic atypical Parkinsonism with dementia. The quantification of the prototypical AAG annonacin in Rat brain homogenates was performed by UPLC-MS/MS in selected reaction monitoring (SRM) mode, using a triple quadrupole mass analyzer. A natural analog of annonacin was used as an internal standard. Analyzed data set of the analytical validation of this method is presented, including stability of the samples, extraction recovery and matrix effect, supporting the results described in the article “Quantification of the environmental neurotoxin annonacin in Rat brain by UPLC-MS/MS” N. Bonneau, I. Schmitz-Afonso, D. Touboul, A. Brunelle, P. Champy (2016) [1].

**Specifications Table**

**Table:** 

Subject area	*Chemistry, Biology*
More specific subject area	*Pharmacokinetic and quantification method*
Type of data	*Tables, figure, text*
How data was acquired	*UPLC-MS/MS: Dionex Ultimate 3000 RSLC system, Triple quadrupole TSQ Vantage EMR*
Data format	*Analyzed*
Experimental factors	*Analysis of Rat brain homogenates, spiked with annonacin*
Experimental features	*Selectivity, carry-over, limits of detection and quantification, accuracy, precision, matrix effect, absolute and relative extraction recovery, stability*
Data source location	*BioCIS, Univ. Paris-Sud, France*
Data accessibility	*Data is with this article*

## Value of the data

• Analyzed dataset for the validation assays of the extraction and quantification of annonacin in the Rat brain is presented.
• Data comprise the analytical parameters determined, including stability, recovery and matrix effect, using UPLC-ESI-triple quadrupole analysis in SRM mode.
• Data are in accordance with the bioanalytical validation criteria set by EMA and can be useful for methodological development.
• They constitute benchmarks for pharmacokinetic studies of sub-type 1b Annonaceous acetogenins.

## 1 Data

The dataset includes the choice of an internal standard and the method validation for the quantification of annonacin, a neurotoxic polyketide, in Rat brain homogenates, by UPLC-ESI(+)-Triple quadrupole. Analyzed data for selectivity, carry-over, limits of detection and quantification, accuracy, precision, matrix effect, absolute extraction recovery, relative extraction recovery and stability are presented.

## 2 Experimental design, materials and methods

### 2.1 Selection of IS

**Fig. 1:**
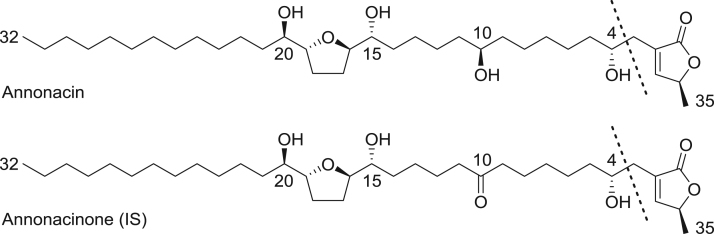
The Annonaceous acetogenins annonacin and annonacinone (internal standard). Product ion spectra are presented in [1].

Annonacin and other AAGs, including isotope-labeled compounds, are not commercially available. Annonacinone (IS) was therefore selected as an Internal Standard (IS), because of its structural similarity with annonacin, resulting in a similar fragmentation pattern [2] with the same specific loss of γ-methyl-γ-lactone (−112 amu). Indeed the structure of IS is identical to that of annonacin except for a ketone instead of a hydroxyl-group at C-10 (Fig. 1). The UPLC-MS/MS analysis conditions were identical to those previously developed for the determination of annonacin in Rat plasma [3]. A full scan MS of annonacin and IS shows that [M+Na]^+^ ions (*m/z* 619.4 and *m/z* 617.4, respectively) were more abundant than [M+H]^+^ ions (*m/z* 597.4 and *m/z* 595.4, respectively). Moreover, only non-specific water losses were observed when fragmenting [M+H]^+^ ions of annonacin and IS, whereas fragmentation of [M+Na]^+^ ions exclusively led to a specific loss of 112 amu, corresponding to the loss of the lactonic ring (Fig. 1). Hence, the SRM mode was used to monitor the transitions *m/z* 619.4 → *m/z* 507.4 and *m/z* 617.4 → *m/z* 505.4, for annonacin and IS, respectively. The [M+2] isotope of IS is also detected when monitoring the transition *m/z* 619.4 → *m/z* 507.4 (Fig. 1B in [1]).

### 2.2 UPLC-MS/MS conditions

Analyses were performed with a Dionex Ultimate 3000 RSLC system equipped with Acquity UPLC BEH C_18_ column (2.1×100 mm, 1.7 µm, Waters, Guyancourt, France) and pre-column (2.1×5 mm, 1.7 µm), under previously described chromatographic conditions [1]. A Triple quadrupole TSQ Vantage EMR (Thermo Scientific, Les Ulis, France) equipped with a heated ESI source was used, in the positive ion mode. The source parameters were set as follows: spray voltage 2000 V, vaporizer temperature 350 °C, sheath gas (N_2_) pressure 45 psi (~3.1 bars), auxiliary gas pressure (N_2_) 20 psi (~1.4 bar), capillary temperature 300 °C. The collision energies were set at 36 eV and 37 eV for annonacin and for the IS, respectively. The data acquisition and processing were performed using Thermo Scientific Xcalibur 2.1 software.

### 2.3 Preparation of calibration standards and quality control samples

Stock solutions of annonacin and IS were prepared at the concentration of 1 mg/mL in methanol and stored at −20 °C. Working standard solutions of annonacin ranging from 5 to 500 ng/mL were prepared daily by successive dilutions of a stock solution in methanol. The stock solution of IS was diluted daily to prepare a working solution at the concentration 100 ng/mL.

The calibration standards were prepared by spiking 1 mL of pooled Rat brain homogenate obtained by sonication in methanol (0.2 g/mL) with 10 μL of IS working solution to reach the concentration of 5 ng/g and with 10 μL of annonacin in solution of appropriate concentration, to reach 0.25; 0.375; 0.5; 1; 2.5; 3.75; 5; 10; 12.5; and 25 ng/g concentrations, respectively. In order to evaluate intra- and inter-day precisions and accuracy, Quality Control (QC) samples were prepared in brain homogenate at the concentrations of 0.25; 0.5; 5; 25 ng/g.

### 2.4 Method validation

The dataset of method validation assay described here comprises selectivity, carry-over, linearity, accuracy, precision, matrix effect, extraction recovery, and stability, according to the EMA guideline on bioanalytical method validation [4].

#### 2.4.1 Selectivity

Data for selectivity determination were obtained on individual brain homogenates. The method was considered selective (absence of interfering component in the matrix) as the response areas at the retention times of annonacin and of IS were respectively less than 20% of the lower limit of quantification (LLOQ) for annonacin, and less than 5% for IS [4], in all runs. For the different blank matrices tested, no interfering compound was detected at the expected retention times of annonacin and IS.

#### 2.4.2 Carry-over

Data for carry-over evaluation were obtained in blank analytical runs following the injection of the upper limit of quantification (ULOQ). The response areas at the retention times of annonacin and of IS were respectively less than 20% of the LLOQ for annonacin and less than 5% for IS, as recommended [4]. No carry-over was observed during the analysis, as assessed after injection of annonacin at ULOQ, neither for IS at working concentration.

#### 2.4.3 Calibration curve and lower limit of quantification (LLOQ)

A calibration curve was designed to cover the expected range of concentration for brain samples [1]. Calibration curves were prepared for each run of analysis, plotting the peak area ratio *y* of annonacin to IS as a function of the nominal concentration of each calibration level *x* and then fitted by linear regression. The LLOQ was defined as the lowest concentration in the calibration curve with a back calculation accuracy expressed as relative error within ±20% and a precision within ±20% [4]. The calibration curves were accepted as back calculated concentrations of the calibration standards were within ±15%.

#### 2.4.4 Accuracy and precision

Data for the evaluation of accuracy and precision were obtained at 4 concentration levels, including LLOQ (0.25 ng/g), low (LQC; 0.5 ng/g), medium (MQC; 5 ng/g) and ULOQ (25 ng/g), with 3 replicates a day, and at two different days. Quality control (QC) samples at 4 different concentrations, including LLOQ and ULOQ, were prepared at 2 different days, with 3 replicates a day, independently of the calibration standards. Within- and between-run precisions were less than ± 15%. Moreover, within- and between-run accuracies were less than ±15% of the nominal concentration (Table 1).

#### 2.4.5 Matrix effect

The matrix effect was investigated by comparison of the peak areas of post-extraction spiked samples at LLOQ and ULOQ, in 3 individual homogenates samples *versus* standard solutions of the same levels. This was performed in triplicate on each homogenate. IS-normalized matrix factor (IS-MF) was calculated. The matrix effect was considered acceptable as the RSD of IS-MF obtained from the 3 individual Rat brains was less than 15%.

A high absolute matrix effect was observed, with signal enhancement for both annonacin and IS. High relative matrix effect was observed at the LLOQ and ULOQ (Table 2). Despite these differences, IS-relative matrix factor evaluated on 3 different brain samples was within 15% at each concentration, as recommended [4].

#### 2.4.6 Extraction recovery

The extraction recovery was evaluated by comparison of the peak areas of extracted samples *versus* post-extraction spiked samples at LLOQ and ULOQ, in triplicate (Table 3). Recovery for annonacin relative to IS, as well as absolute recoveries for annonacin and IS are presented. Absolute recovery was very low (5–10%). However, the relative recovery was reproducible, with a maximal error of 15%, calculated as: *Δ*_(*BE*/*PE*)_= 100*(mean_PE_*SD*_BE_*+mean_BE_*SD*_PE_*)/(mean*_PE_*)^2^; where *BE* is the area before extraction and *PE* the area after extraction.

#### 2.4.7 Stability

The stability of annonacin during sample processing and storage was evaluated in triplicate at room temperature during 4 h, under three freeze-thaw cycles, and during 24 h on the autosampler at 4 °C. Analysis performed in triplicate at three concentration levels, LLOQ, MQC and ULOQ, showed variations within ±15% of their nominal concentration, evidencing no loss of annonacin during the process (Table 4).

## Figures and Tables

**Table 1:** Precision and accuracy for the determination of annonacin in Rat brain (*n*=3).

**Concentration(ng/g)**	**Within-run**			**Between-run**		
	**Pecision**	**Accuracy %**		**Precision**	**Accuracy %**	
	**(RSD %)**	**Mean**	**±SD**	**(RSD %)**	**Mean**	**±SD**
0.25	10.0	113.9	8.0	9.0	114.3	10.3
0.50	5.8	105.8	7.9	5.6	102.6	5.8
5	8.3	93.1	1.1	3.3	100.6	9.0
25	3.4	90.1	4.5	14.0	100.2	11.4

**Table 2:** Evaluation of matrix effect on 3 individual Rat brain homogenates (mean±RSD%).

***n*=3 individual brain homogenates**	**Concentration (ng/g)**	**Matrix factor %**		**IS-normalized matrix factor %**	
**Mean**	**±RSD**	**Mean**	**±RSD**
Annonacin	0.25	502	6	294	6
25	279	13	148	4
IS	5	171	4		

**Table 3:** Extraction recovery of annonacin and IS (*n*=3, mean ± ∆_max_ %).

***n*=3**	**Concentration (ng/g)**	**Absolute recovery %**		**Relative recovery %**	
**Mean**	**± Δ**_**max**_	**Mean**	**± Δ**_**max**_
Annonacin	0.25	5.0	1.2	56.2	7.8
25	10.7	1.5	66.1	4.2
Annonacinone (IS)	5	7.2	2.6		

**Table 4:** Stability of annonacin in Rat brain homogenates (*n*=3).

***n*=3**	**Concentration (ng/g)**	**% of nominal concentration**	
**Mean**	**±SD**
4 h RT	0.25	110	7
25	93	3
3 cycles −20 °C - RT	0.25	108	5
25	92	5
24 h autosampler 4 °C	0.25	102	2
25	91	1
